# Application of next-generation sequencing for the high-resolution typing of MHC-*B* in Korean native chicken

**DOI:** 10.3389/fgene.2022.886376

**Published:** 2022-10-28

**Authors:** Thisarani Kalhari Ediriweera, Prabuddha Manjula, Eunjin Cho, Minjun Kim, Jun Heon Lee

**Affiliations:** ^1^ Department of Bio-AI Convergence, College of Engineering, Chungnam National University, Daejeon, Korea; ^2^ Department of Animal Science, Uva Wellassa University, Badulla, Sri Lanka; ^3^ Division of Animal and Dairy Science, College of Agriculture and Life Sciences, Chungnam National University, Daejeon, Korea

**Keywords:** assembly, Korean native chicken, MHC-*B*, NGS, variants

## Abstract

The major histocompatibility complex-*B* (MHC-*B*) region of chicken is crucially important in their immunogenesis and highly diverse among different breeds, lines, and even populations. Because it determines the resistance/susceptibility to numerous infectious diseases, it is important to analyze this genomic region, particularly classical class I and II genes, to determine the variation and diversity that ultimately affect antigen presentation. This study investigated five lines of indigenous Korean native chicken (KNC) and the Ogye breed using next-generation sequencing (NGS) data with Geneious Prime-based assembly and variant calling with the Genome Analysis Toolkit (GATK) best practices pipeline. The consensus sequences of MHC-*B* (*BG1*-*BF2*) were obtained for each chicken line/breed and their variants were analyzed. All of the Korean native chicken lines possessed an excessive number of variants, including an ample amount of high-impact variants that provided useful information regarding modified major histocompatibility complex molecules. The study confirmed that next-generation sequencing techniques can effectively be used to detect MHC variabilities and the KNC lines are highly diverse for the MHC-*B* region, suggesting a substantial divergence from red junglefowl.

## Introduction

The major histocompatibility complex (MHC) of chicken comprises two genetically disassociated gene clusters, MHC-*B* and MHC-*Y*, and the MHC-*B* contains the minimal essential region. It is largely responsible for the immune response and histocompatibility of chicken. It possesses classical MHC class I and II genes that produce MHC class I and class II molecules, which are extremely important biological agents in the recognition of foreign pathogenic peptides. It also carries BG family genes and C-type lectin like loci (Kaufman et al., 1999; Shiina et al., 2007; Kaufman, 2021).

The *BF1* and *BF2* genes belong to classical class I of the minimal essential region, while the *BLB1* and *BLB2* genes are in classical class II. Class I and II genes are extremely important in adaptive immune response, because they are recognized by cytotoxic and helper T lymphocytes, respectively; some are also important in innate immunity (i.e., the *BF1* gene, the minor class I gene that corresponds with natural killer cells as ligands). The *BG1* gene is also involved in adaptive immune response (Goto et al., 2009; Chen et al., 2018; Kaufman, 2021).

Numerous serological and molecular biological techniques have been used to study the MHC diversity of chicken. Although low-resolution MHC typing methods are capable of providing insights regarding diversity and haplotypes, they are still not able to describe the region as a whole, particularly class I and II variation (Fulton, 2020). Hence, the MHC haplotypes identified from different genetic sources based on single-nucleotide polymorphism (SNP)/microsatellite markers or direct sequencing need to be validated by high-resolution sequence typing to better understand the region.

Currently, next-generation sequencing (NGS) approach can generate the full-length sequences of highly polymorphic genomic regions such as MHC, with extremely high coverage and precision at each variant. Target polymerase chain reaction (PCR) and valid sequence information of the BF/BL region are crucial for haplotype identification and nomenclature.

A PCR-NGS approach for MHC haplotype identification has been developed and successfully applied in human leukocyte antigen (HLA) typing (Ozaki et al., 2015; Ka et al., 2017; Alizadeh et al., 2020). However, there are limited chicken MHC sequence data available with a sufficient sample count to describe the enormous haplotype diversity due to the forces of recombination and gene conversion within the MHC region. The databases containing such data were generated solely from MHC-*B* homozygous, inbred chicken lines. Local chicken breeds have not undergone such intensive selection for MHC polymorphism, which makes it difficult to obtain homozygous individuals. Recently, many DNA-based typing methods have been developed and successfully applied to local chickens of various origins (Fulton et al., 2016; Mwambene et al., 2019; Manjula et al., 2020). This difficulty can therefore be overcome by collecting primary data from low-resolution typing methods and then generating reliable nucleotide sequence data from high-throughput sequencing such as NGS.

The term “Korean native chicken” (KNC), as used in this study, comprises two major native chicken breeds: the KNC and Ogye chicken. The KNC breed carries five lines of gray, black, red, white, and yellow. The Ogye breed is also native to Korea and considered part of the national heritage. Hereafter, they are collectively referred to as six KNC lines.

These KNC lines are becoming increasingly popular due to their characteristic flavor and high meat quality (Manjula et al., 2020; Nawarathne et al., 2020). Although they were highly valued by Koreans in the past, their populations were substantially decreased during the Korean war. Based on collaborative efforts by the Korean government, scientific community, and livestock farmers, there is now a trend towards their commercial farming. Because disease resistance plays a vital role in chicken farming, it is very important to investigate the MHC region of KNC to prepare them as commercial breeds with a high disease resistance. Previous MHC studies on KNC lines have shown that they have a unique MHC diversity, while also share a few common haplotypes with commercial chicken breeds (Manjula et al., 2020). The presence of homozygous individuals with novel haplotypes in KNC pure line populations creates an opportunity to further investigate their haplotype diversity by developing reliable sequence information from a large section of MHC-*B*.

We analyzed six novel BSNP-MHC haplotypes (Manjula et al., 2020) of six KNC lines that are homozygous for both SNP and microsatellite markers, using long-range PCR and NGS methods. The consensus sequences of these novel BSNP haplotypes provide reference data to better understand new variants in KNC. Accordingly, an NGS-based MHC typing technique could be developed for local chicken breeds.

## Materials and methods

### DNA samples

Six birds, one each from six KNC lines, homozygous for both LEI0258 microsatellite marker and 90 MHC-*B* SNP panel described in our previous study (Manjula et al., 2020), from the National Institute of Animal Science (South Korea) were used. Before the investigation began, the birds were further analyzed for chromosome 16, which contains MHC genes, using the 600 K SNP chip to assess their MHC class I and class II variants (unpublished data). Birds that were homozygous for all markers were accepted for sequencing.

Haplotypes distinguished by the SNP panel and microsatellite typing included BSNP-B03 (249/249), BSNP-Kr11 (193/193), BSNP-Kr15 (193/193), BSNP-J06 (474/474), BSNP-B03 (249/249), and BSNP-Kr31 (417/417) from the Korean gray, black, red, white, yellow, and Ogye lines, respectively (Manjula et al., 2020). Genomic DNA was extracted as described previously (Manjula et al., 2020).

### Long-range PCR (LR-PCR) amplifications

A total of 16 pairs of primers were used, including 10 LR-PCR pairs described in Hosomichi et al. (2008) and six new pairs designed using the Prime3 Software available on the National Center for Biotechnology Information (NCBI) website (https://www.ncbi.nlm.nih.gov/tools/primer-blast/index.cgi?LINK_LOC = BlastHome). They were used for the LR-PCR amplifications of 15 MHC genes (*BG1*—*BF2*) belonging to the extended class I, classical class I and class II, covering approximately 69 kb of the chicken MHC-*B* region.

Initially, all 16 PCR products were amplified using LR-PCR primers designed based on the MHC-*B* reference sequence AB268588.1 (Shiina et al., 2007). All of the LR-PCR amplifications were carried out using Takara PrimeSTAR polymerase (Takara Bio, Japan) based on optimized two-step and three-step standard protocols (Cat. #R050A). In brief, the 20 µL PCR reaction volume included 100 ng template DNA, 1 µL (10 pmol) of each forward and reverse primers, 4 µL 5 × GL buffer, 1.6 µL dNTP, 0.4—0.5 µL Prime STAR polymerase, and distilled water. The size of the LR-PCR was 4,780 kb on average, with a range from 1,345 to 9,437 bp (Supplementary Table S1).

To measure the success of LR-PCR amplifications, PCR products were checked on a 0.8% agarose gel and visualized by staining with ethidium bromide. The PCR products were purified using a PCR purification kit (Genet Bio, Daejeon, South Korea) and the concentrations were obtained using a NanoDrop device (Thermo Fisher Scientific, NanoDrop, 2000C). The normalization of PCR product concentration and PCR product pooling were conducted based on the equimolar pooling method. Pooled PCR products of BSNP-Kr11 (193/193) from the black line were sent to the TNT research facility (South Korea) for library preparation and NGS.

After obtaining the NGS results, areas containing gaps or ambiguities were identified by the mapping procedure described later in the methodology. To further optimize the results for the remaining five lines, seven new LR-PCR primer pairs were prepared based on AB268588.1 (Shiina et al., 2007) and the NGS consensus of the BSNP-Kr11 (193/193) sample. Accordingly, a total of 23 LR-PCR products were obtained for the remaining five samples following the same PCR amplification protocol.

### Library preparation and NGS for PCR amplicons

Purified and pooled PCR products were sent for NGS. After performing DNA quality control using PicoGreen (cat.#P7589; Invitrogen) and the DNA High Sensitivity Chip (Bioanalyzer), qualified samples were used for library construction using a TruSeq DNA PCR-Free (350) kit, according to the TruSeq DNA PCR-Free Sample Preparation Guide (Part #15036187 Rev. D) and sequenced with the 6000 S4 Reagent kit (ver. 1.5; NovaSeq; 300 cycles) using the Illumina NovaSeq platform.

### NGS data pre-processing and assembly

The NGS reads were assembled using the Geneious Prime molecular biology tools and subjected to sequence analysis using RRID:SCR_010519 (ver. 2022.0.1; Geneious).

The raw NGS reads were imported into the Geneious Prime tool (Illumina read technology), and paired reads were set with the relative orientation of forward/reverse inward-pointing (Illumina paired-end) with an insert size of 350 bp. Then, they were trimmed and normalized using BBDuk Adapter/Quality trimmer (version 38.84) and BBNorm (version 38.84), respectively.

In the trimming process, the default parameters were right end trimming with a K-mer size of 27, with the maximum substitutions set to one and maximum ‘substitutions + indels’ set to 0. The low-quality bases, i.e., quality lower than 20, were trimmed from both ends and the minimum overlap was set to 20 when trimming adapters on paired read overhangs. Short reads with a maximum length of 10 bp were discarded. Two-pass normalization was conducted by adhering to the default settings where the target coverage level/target normalization depth and minimum depth were adjusted to 40 and 6, respectively. The number of threads was set to 12 while the K-mer size was 31.

The *Gallus* genes, MHC region, and partial and complete coding sequences (CDSs) (accession number: AB268588.1) available in the NCBI and published by Shiina et al. (2007) were used as the reference and annotated directly against the NCBI database using the Geneious Prime GenBank accession tools for both genes and CDSs. Then the targeted MHC-*B* region was extracted.

Next, the normalized reads were assembled with the “map to reference” technique using Geneious mapper under medium sensitivity, as per the instructions provided in the product manual. Paired read overhangs were trimmed and gaps were allowed (maximum per read = 15%). However, trimming from the Geneious mapper prior to the mapping was restricted because the trimming was conducted with BBDuk in a previous step. The program was set to generate contigs, a consensus sequence, and an assembly report for each assembly. Once the assembly was completed, the consensus sequences were obtained as the DNA sequence for the relevant portion of MHC-*B* for each of the KNC lines separately. Then they were computationally annotated using the reference-based technique.

The same raw NGS data were analyzed again with the GATK best practices pipeline to obtain the variants. For this pipeline, the chromosome 16:GRCg6a:16:1:2844601:1, which is available in the Ensembl genome browser, was set as the reference (Ensembl.com, 2021). Then the variants were analyzed using the pandas library in Python.

## Results

### LR-PCR amplifications

Once the extracted DNA from the selected individuals of six KNC lines were amplified with the primers as described previously (targeting the *BG1*—*BF2*), the LR-PCR amplified products were checked *via* agarose gel electrophoresis. (Images for all six lines are shown in Supplementary Figure S1). The results confirmed that the products for all six lines were successfully amplified and had the expected fragment sizes.

### NGS for PCR amplicons

The NGS reads were paired-end, with sequencing read length and insert size of 151 and 350 bp, respectively. Details of the sequencing results are presented in Table 1.

**TABLE 1:** Raw NGS data and assembly statistics for six KNC lines with known and novel variants obtained for the MHC-*B* region (variants with an Ensembl-based rs-number were considered known variants).

Feature		KNC line					
		Black	Gray	Red	White	Yellow	Ogye
Total read bases		1,468,886,324	2,244,799,220	2,261,956,444	2,199,870,982	2,127,926,126	2,155,747,272
Total reads		9,727,724	14,866,220	14,979,844	14,568,682	14,092,226	14,276,472
GC (%)		59.54	60.26	60.28	60.23	60.74	60.42
Length (bp)		69,269	72,456	72,680	69,069	71,522	69,190
Identical sites		55,749	43,914	44,371	44,264	43,870	44,316
Pairwise identity (%)		81.5	70.8	75.1	70.1	70.2	85.6
Known variants (*BG1* – *BF2*)	SNPs	477	402	427	484	428	373
	Indels	10	3	4	3	5	5
Novel variants (*BG1* – *BF2*)	SNPs	92	185	148	106	108	132
	Indels	69	59	71	65	61	57
Total number of variants (*BG1* – *BF2*)		648	649	650	658	602	567

The use of a lesser number of primer pairs to produce PCR products resulted in a lesser number of total reads and read bases in the black line than the other five lines. The average total reads and read bases of the KNC lines except for the black line were 14,556,689 and 2,198,060,009, respectively. In all cases, the guanine-cytosine (GC) contents were between 59.54 and 60.74, whereas the adenine-thymine (AT) contents were close to 40%.

## Pre-processing and assembly of NGS data

### Assembly with Geneious Prime

All six lines resulted in read recoveries in excess of 93% after trimming, with the lowest value obtained for the yellow line (93.46%), and the highest obtained for the black line (96.58%). The reason behind the recovery of the highest percentage of reads after trimming in the black line may be due to the lesser number of reads generated compared to the other five lines, which was a consequence of the comparatively low number of primer pairs used.

When assembling the reads to the targeted MHC-*B* region of AB268588.1 (74,072 bp), the following statistics were obtained (Table 1). The numbers of identical sites between the KNC lines and the reference (AB268588.1) ranged from 43,870 in the yellow line to 55,749 in the black line. Furthermore, the pairwise identity percentages ranged from ∼70% to ∼86%, revealing noticeable deviations of KNC from the reference. An example of the visualization of a completed assembly, annotations, and the consensus sequence is given in Supplementary Figure S2.

Based on the Lander–Waterman equation (Lander and Waterman, 1988), the coverages of the assemblies for the KNC gray, black, red, white, yellow and Ogye breeds were approximately 146, 105, 138, 136, 136, and 123, respectively.

After their annotations, all of the lines were called for all of the expected genes, often with varying lengths due to insertions and deletions (indels; Table 2). The size of *Blec4* pseudogene (Shiina et al., 2007; Yuan et al., 2021) was the same for the reference and all six lines. In the case of *TAP2*, all KNC lines had a size of 2997 bp, unlike the reference *TAP2* (3037 bp). The same pattern was observed for *BRD2* (3,763 bp in all KNC lines vs. 3,762 bp in the reference). The sizes of the other 12 genes differed from the reference, at least in one KNC line.

**TABLE 2:** Summary of the sizes of MHC-*B* genes (from *BG1*-*BF2*) in KNC lines.

Gene	Reference (AB268588.1)	KNC line					
		Black	Gray	Red	White	Yellow	Ogye
*BG1*	4267 bp	4266 bp	4266 bp	4212 bp	4268 bp	4266 bp	4266 bp
*Blec4*	720 bp	720 bp	720 bp	720 bp	720 bp	720 bp	720 bp
*Blec2*	2493 bp	2490 bp	2481 bp	2490 bp	2543 bp	2481 bp	2484 bp
*Blec1*	2074 bp	2073 bp	2074 bp	2074 bp	2073 bp	2074 bp	2074 bp
*BLB1*	1364 bp	1357 bp	1359 bp	1364 bp	1364 bp	1358 bp	1365 bp
*TAPBP*	3450 bp	3443 bp	3445 bp	3443 bp	3449 bp	3445 bp	3452 bp
*BLB2*	1352 bp	1352 bp	1358 bp	1341 bp	1353 bp	1353 bp	1341 bp
*BRD2*	3762 bp	3763 bp	3763 bp	3763 bp	3763 bp	3763 bp	3763 bp
*DMA*	2087 bp	2088 bp	2089 bp	2089 bp	2087 bp	2089 bp	2089 bp
*DMB1*	1820 bp	1819 bp	1822 bp	1821 bp	1821 bp	1822 bp	1821 bp
*DMB2*	2937 bp	2982 bp	2938 bp	2957 bp	2946 bp	2945 bp	2946 bp
*BF1*	2034 bp	2023 bp	2009 bp	2024 bp	2013 bp	2024 bp	2032 bp
*TAP1*	4797 bp	4835 bp	4799 bp	4799 bp	4808 bp	4799 bp	4808 bp
*TAP2*	3037 bp	2997 bp	2997 bp	2997 bp	2997 bp	2997 bp	2997 bp
*BF2*	2017 bp	2014 bp	2008 bp	2022 bp	2019 bp	2017 bp	2017 bp

### Variant calling with the GATK pipeline

The NGS reads were processed with the GATK pipeline in which the variants were called and annotated with SnpEff. The results were analyzed and simplified to aid understanding. We initially analyzed the known and novel variants for each line. The results are presented in Table 1.

The numbers of variants were 649, 648, 650, 658, 602, and 567 for the gray, black, red, white, yellow, and Ogye lines, respectively. All six KNC lines contained both known and novel variants. Accordingly, 37.6%, 24.9%, 33.7%, 26.0%, 28.1%, and 33.4% of the total variants were novel in the gray, black, red, white, yellow, and Ogye lines, respectively. More than 57% of them were SNPs (ranging from 57.2% in the black line to of 75.9% in the gray line) and others were indels.

The variants were further categorized by SnpEff (within the GATK pipeline) based on their impacts on protein synthesis. When the SnpEff analysis resulted in two or more transcripts for the same genes, the Ensembl canonical transcript was called for the gene of concern.

**FIGURE 1:**
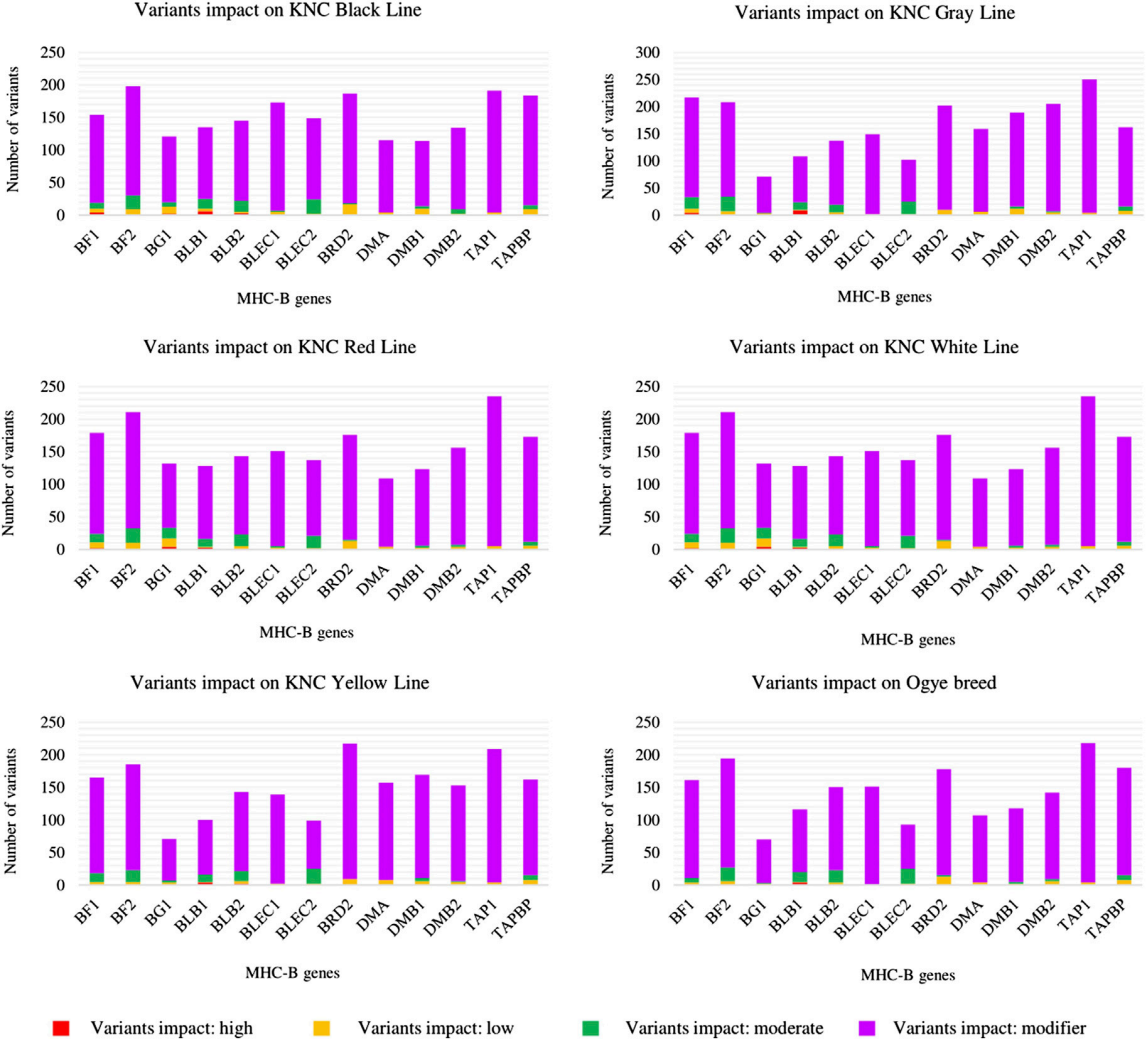
Impact of the variants on MHC-*B* genes for six KNC lines.

The genes in the MHC-*B* region (from *BG1* to *BF2*) were analyzed in terms of the impact of variants for each line (Figure 1 and Supplementary Table S2). The high-impact variants that caused immensely disruptive changes/modifications in the respective proteins could be found for only a few of the most important genes (*BF1*, *BF2*, *BLB1*, *BLB2*, and *BG1*) in all six lines.

*BLB1*, which is a minor class II gene (Jacob et al., 2000), was the only gene with high-impact variants in all six KNC lines. Other genes (*BF1*, *BF2*, *BLB2*, and *BG1*) carried high-impact variants only in some lines. Furthermore, *BLB1* had the most high-impact variants in all KNC lines, except for the red and white lines. Considering all impact types, *BLB2* always led *BLB1* in terms of the number of variants, confirming the higher number of polymorphisms in *BLB2*.

The other genes in which no high-impact variant was found did, however, derive moderate-, low-, and modifier-impact variants. Based on the impact variants (considering only the canonical transcripts), the fewest variants obtained for a single gene of a single line was recorded for *BG1* of the Ogye breed (70 variants), while the most was recorded for *TAP1* of the KNC gray line (250 variants).

For the variant types, upstream, downstream, or intron variants were obtained in the first three places based on the number of variants in each of the lines with different orders. The least number of variants per line was given by one or more of the following variant types: conservative inframe deletion, disruptive inframe insertion, disruptive inframe deletion, stop gain, 5′UTR premature start codon gain, splice donor, and splice acceptor frameshift. Stop gains were comparatively rare and accounted for the high-impact variants, while all lines had missense variants with moderate impacts.

## Discussion

The MHC region in the chicken plays a key role in adaptive and innate immunity; in this region, the BF/BL genes are predominantly involved in antigen presentation, and therefore determine the resistance/susceptibility to various diseases, such as Marek’s disease (Kaufman et al., 1999) and avian influenza (Hunt et al., 2010).

NGS approaches have been applied in avian species that are closely related to chickens, such as quail (Kawahara-Miki et al., 2013) and turkeys (Dalloul et al., 2010), to study such variation. Although many scientists are currently working with NGS for chicken MHC analysis, to our knowledge, no previous study on the utility of LR-PCR combined with NGS for chicken MHC genotyping has been published.

When assembling the pre-processed NGS reads by mapping to the AB268588.1 reference, it was first directly annotated to the NCBI source data, particularly for its genes and CDSs. One of the annotation notes is shown in Supplementary Figure S2. This facilitated an easy extraction of the target reference region, thus improving assembly quality. The average pairwise identity between the assemblies of KNC lines and the reference provides evidence of a possible evolutionary divergence among them with respect to MHC-*B*.

As MHC class I and class II genes obtain significant importance among the MHC genes, some observations about them are highlighted. Among the MHC-class I genes, *BF2* is dominantly expressed (Parker and Kaufman, 2017) and highly polymorphic (Shaw et al., 2007). Considering the Ensembl canonical transcripts, *BF2* always had more variants than *BF1*, except for the gray line. We observed more polymorphisms in *BF1* than *BF2* in the KNC gray line, based on their canonical transcripts (Supplementary Table S2).

Because *BLB1* and *BLB2* are responsible for producing the MHC class II molecules, they are also vital for activating/initiating adaptive immunity in chicken (Worley et al., 2008; Kaufman, 2021). The exon two of both *BLB1* and *BLB2* contributes to the fabrication of the peptide-binding groove/peptide binding region of the MHC class II molecules. Therefore, the polymorphisms in that region cause substantial changes in peptide binding affinity (Li et al., 2010). This study confirms the high number of polymorphisms in both MHC class II genes, and provides an insight into the modified form of MHC class II molecules in KNC lines compared to the reference red junglefowl.

These high-impact variants in highly polymorphic, functional, and important MHC genes provide clear insight of the differentiated immune responses in KNC lines compared to the reference red junglefowl. However, the actual effects of such variants on protein modifications are yet to be discovered, and it is not possible to comment on their benefits or drawbacks in effective immune responses.

Our data (Supplementary Table S2) showed that several variant types occur throughout the genes in all lines. These results suggest a very high diversity in KNC lines compared to the reference red junglefowl.

Consequently, the NGS technique can be used to reliably detect MHC-*B* variabilities, in contrast to some of the previously used marker-based methods. However, NGS reads with larger insert sizes may greatly increase the assembly quality by increasing the probability of alignment at the most accurate locations, and we therefore recommend the use of such NGS techniques.

## Conclusion

We investigated a portion of the chicken MHC-*B* region (from *BG1* to *BF2*), including the classical class I and II genes, completely based on the NGS data of the six KNC lines. After quality control and assembly, their consensus sequences were derived from *BG1* to *BF2* for all six KNC lines. The variants were analyzed and numerous novel variants were revealed. The results indicated that all of the KNC lines were highly diverse and diverged from the reference red junglefowl.

## Data Availability

The datasets presented in this study can be found in online repositories. The names of the repository/repositories and accession numbers can be found below: https://www.ncbi.nlm.nih.gov/genbank/, OM953772, OM953773, OM953774, OM953775, OM953776, and OM953777.
