# Supplementary material for: Application of next-generation sequencing for the high-resolution typing of MHC-B in Korean native chicken
Source: Front Genet. 2022 Oct 28;13:886376. doi: 10.3389/fgene.2022.886376 (PMC9649677; doi:10.3389/fgene.2022.886376)
Supplement: Supplementary file 2 [file Table1.docx]

Supplementary Table 1. Target region, primer pair, amplified length, and long-range PCR conditions for selected samples

| Product | Region | Primer name | Primer (5'-3') | Physical position^1^ | | Amplified  length (bp) | PCR condition | |
| --- | --- | --- | --- | --- | --- | --- | --- | --- |
|  |  |  |  | From | To |  | Procedure | Extension time (minutes) 1min/kb |
| P1 | BG1 | BG1_F1 | CTCAGTTCGGTGGTTTCTCAGG | 128863 | 133887 | 5,024 | Two step^1^ | 6 |
|  |  | BG1_R1 | GCTGCAATGGGATGTGTTTTATG |  |  |  |  |  |
| P2 | Blec4^a^ | BLEC4_F | CCGGTGCTGGATCCATCTGATGTCTG | 132711 | 137161 | 4,450 | Three-Step^2^ | 5 |
|  |  | BLEC4_R | TGGAGGGCGACGTCTGTAGTGGTGAA |  |  |  |  |  |
| P3 | Blec2 ^a^ | Blec2_F2 | CAGAACCGCTGCTTTCCTTGCACA | 136614 | 140973 | 4,359 | Two step | 5 |
|  |  | Blec2_R3 | GTCCGTCTGATGCTGAATGCGAAAAT |  |  |  |  |  |
| P4 | Blec1 ^a^ | Blec1_NF3 | CATCATCTGCTTCATTTCCAATTCCCC | 140397 | 143994 | 3,598 | Two step | 5 |
|  |  | Blec1_R1 | TTCCTGTGACAAAACACCGTTAAAAGTGA |  |  |  |  |  |
| P5 | BLB1 ^a^ | BLB1_SF2 | GGCGGAGCTTCGGCTCCAAATTACAT | 143666 | 146087 | 2,421 | Two step | 5 |
|  |  | BLB1_SR2 | CATGAGGGGATCATGAAGGGGCAGAG |  |  |  |  |  |
| P6 | BLB1-TAPBP ^a^ | BLB1_SF3 | TCACCCGTCTCCAGGCAGAGTT | 145811 | 147164 | 1,353 | Two step | 5 |
|  |  | BLB1_SR3 | GACAATGGGAGATCATGGATTTGGGT |  |  |  |  |  |
| P7 | Tapasin ^a^ | Tapasin_F1 | TCTACCTGGACTTGTGTGCGGCCATT | 146522 | 150713 | 4,191 | Two step | 5 |
|  |  | Tapasin_R2 | ACTCATCTGCCTCACCAACCCAGGA |  |  |  |  |  |
| P8* | TAPBP | BL2_ST2_F1 | ATGGACGCACCAAAGACGTG | 147958 | 152668 | 4,711 | Two step | 5 |
|  |  | BL2_ST2_R1 | AGCGCGTTTATTAGGATGGGGAC |  |  |  |  |  |
| P9 | BLB2 ^a^ | BLB2_F2s | CTCTCCGCTGCTTTCGCTTT | 150146 | 152630 | 2,484 | Two step | 5 |
|  |  | BLB2_R3s | GCGACATCTCCAACGGACG |  |  |  |  |  |
| P10* | BRD | BL2_ST2_F2 | AATAAACGCGCTGACTTTGACC | 152656 | 155213 | 2,559 | Two step | 5 |
|  |  | BL2_ST2_F2 | TTGAGTTCAGCTACGCCAAGAT |  |  |  |  |  |
| P11 | BLB2_BRD | BL2_BR_N_F | CTAATGAATGAAGTGGACAGGGTCT | 151675 | 157055 | 5,379 | Two step | 6 |
|  |  | BL2_BR_N_R | ATCTTGTGACCCCAATATTAAACGC |  |  |  |  |  |
| P12 | BRD2-DMA | LP3_F | TGCATAGGGGATGGACAGTCAG | 156622 | 163,500 | 6878 | Three-Step | 5 |
|  |  | LP3_R | CCTTGCACAACTTCTTGCCCCAGT |  |  |  |  |  |
| P13 | DMA-DMB1 | DMA_DB_N_F1 | TAGAGCTGCTTATTAATTGCTTGCG | 163025 | 168454 | 4969 | Two step | 6 |
|  |  | DMB1_B2_N_R2 | CCTCAATATTGCAAAGGCTGAGAC |  |  |  |  |  |
| P14* | DMA | DMA_ST2_F1 | AAACCCAATGCCTGAAGCCAAG | 164039 | 169688 | 5,650 | Two step | 6 |
|  |  | DMA_ST2_R1 | CGTCCGATCCTCCATACAACAT |  |  |  |  |  |
| P15 | DMB2 ^a^ | DMB2_F | CTCCTCATTCATCATCTTCTGTGTG | 167504 | 171345 | 3,841 | Two step | 6 |
|  |  | DMB2_R | ATGACTCATGACCTATCCAGATCAC |  |  |  |  |  |
| P16* | BF1 | DMB_ST2_F1 | TTCAACAAGAACCCTCTGGTGT | 169026 | 173722 | 4,696 | Two step | 6 |
|  |  | DMB_ST2_R1 | TGGATCCACCTTCCCTGTCTAT |  |  |  |  |  |
| P17* | BF1 | BF1_ST2_F1 | GATGGACACCTTCTGTCACCAA | 171062 | 175291 | 4,229 | Two step | 6 |
|  |  | BF1_ST2_R1 | GACATCCCCCTGACCGTTTCTC |  |  |  |  |  |
| 18* | TAP1 | TAP1_ST2_F1 | CGTTCTGCTTCCTATGCTGACT | 174007 | 175352 | 1,345 | Two step | 5 |
|  |  | TAP1_ST2_R1 | CCTGATCTGGTGTCCCCTTATCT |  |  |  |  |  |
| 19* | TAP1 | TAP1_ST2_F2 | GTGGAGATAAGGGGACACCAGAT | 175325 | 180,279 | 4,954 | Two step | 6 |
|  |  | TAP1_ST2_R2 | ACACAGCAGTGCTTTTAGAACG |  |  |  |  |  |
| P20 | TAP1^a^ | TAP1_F2 | GGGTAAGGATGGGTATAGGCTGGGCACT | 174821 | 179547 | 4,727 | Two step | 6 |
|  |  | TAP1_R1 | CAGTGGCACCGCAGTCCATGC |  |  |  |  |  |
| P21 | TAP2-BF2 | TAP2__N_F | GGTGAACCTCTGAAGCTCATTGTTG | 179144 | 188581 | 9,437 | Two step | 10 |
|  |  | BF2_N_R | AACATTCATAGTCCCATAGCTACCC |  |  |  |  |  |
| P22* | TAP2-BF2 | BF2_ST2_F1 | CTCCTATGGTGACCTTCTGAGC | 182383 | 188216 | 5,833 | Two step | 6 |
|  |  | BF2_ST2_F1 | AGCCTCTAACCCTATGGATGTGA |  |  |  |  |  |
| P23 | BF2-C4 | BF2_C4_F | CTTCCTTTGAGATCAGTGCCATTTC | 188260 | 197657 | 9,397 | Two step | 10 |
|  |  | Bf2_C4_R | CAAACACCATAGAGAGATGCAATCC |  |  |  |  |  |

All the new primers were prepared based on the sequence ID: AB268588.1

*New LR-PCR primers prepared based on the new NGS consensus of Black line.

^a^ LR-PCR primers reported in Hosomichi et al. (2008).

^1^ Two-step PCR condition: 32 cycles of 98 ̊C 10 sec, 68 ̊C, 5-6 minutes (based on the product size)

^2^ Three-step PCR condition: 32 cycles of 98 ̊C 10 sec, 60 ̊C, 18 sec, 68 ̊C, 5 minutes (based on the product size)
