# Supplementary material for: Application of next-generation sequencing for the high-resolution typing of MHC-B in Korean native chicken
Source: Front Genet. 2022 Oct 28;13:886376. doi: 10.3389/fgene.2022.886376 (PMC9649677; doi:10.3389/fgene.2022.886376)
Supplement: Supplementary file 3 [file Presentation1.pdf]

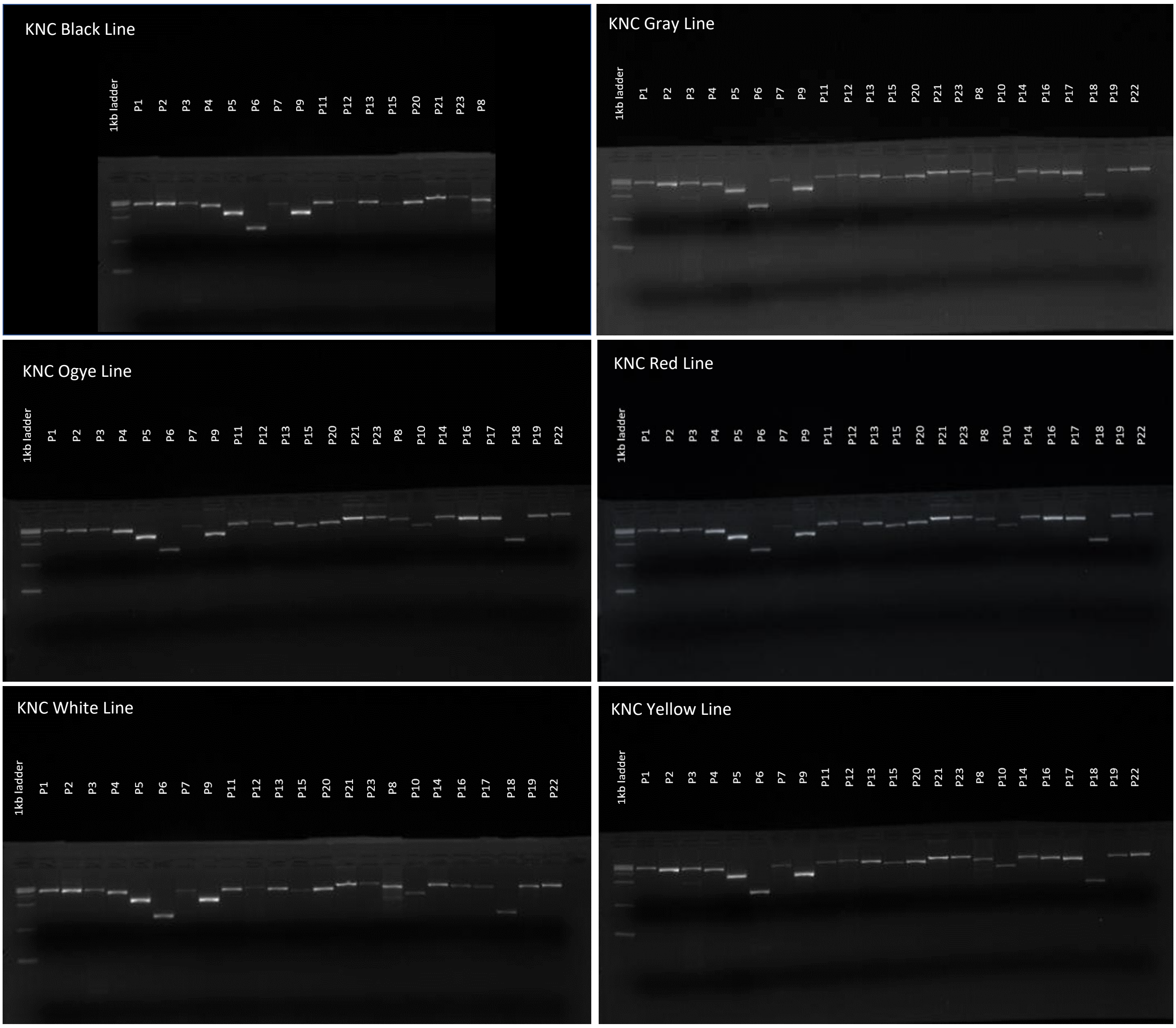

Supplementary Figure 1. Agarose gel electrophoresis images of long-range PCR amplification of MHC-*B* region for all the six KNC lines (23 primer pairs have been used to amplify the MHC-*B* region of KNC lines except the black line (the first line sequenced)

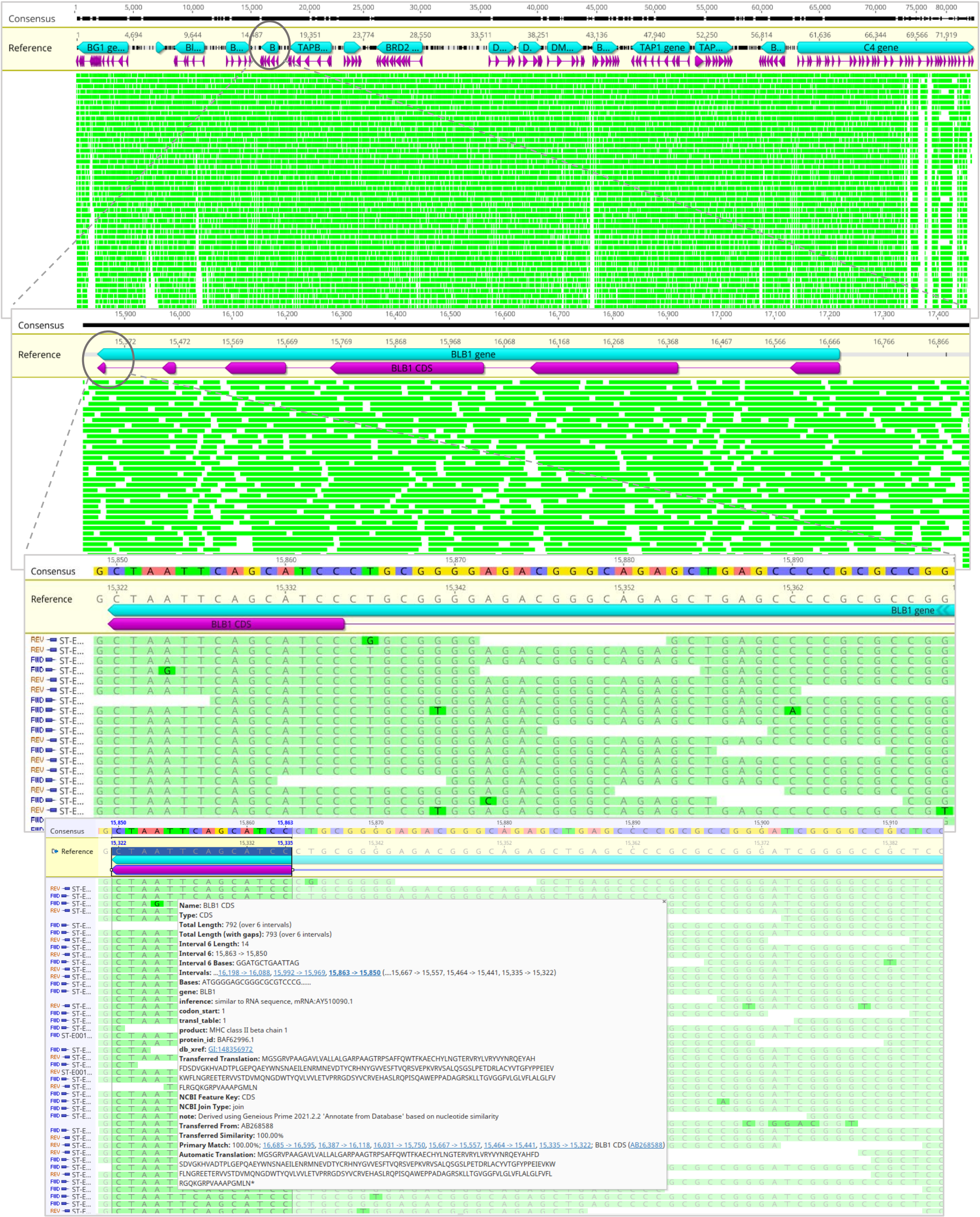

Supplementary Figure 2. An example for a visualization of completed assembly, annotations and the consensus sequence of MHC-*B* region of KNC.
